# The emerging role of deubiquitinating enzymes in genomic integrity, diseases, and therapeutics

**DOI:** 10.1186/s13578-016-0127-1

**Published:** 2016-12-20

**Authors:** Mingjing He, Zhuan Zhou, Anil A. Shah, Haojing Zou, Jin Tao, Qianming Chen, Yong Wan

**Affiliations:** 1 Department of Cell Biology, University of Pittsburgh School of Medicine, 5117 Centre Avenue, Hillman Cancer Center, HCC2.6c, Pittsburgh, PA 15213 USA; 2 State Key Laboratory of Oral Diseases, West China Hospital of Stomatology, Sichuan University, Chengdu, 610041 Sichuan People’s Republic of China

**Keywords:** Deubiquitinases, DNA damage response, DNA damage repair, Tumorigenesis, Anti-cancer treatment

## Abstract

The addition of mono-ubiquitin or poly-ubiquitin chain to signaling proteins in response to DNA damage signal is thought to be a critical event that facilitates the recognition of DNA damage lesion site, the activation of checkpoint function, termination and checkpoint response and the recruitment of DNA repair proteins. Despite the ubiquitin modifiers, removal of ubiquitin from the functional proteins by the deubiquitinating enzymes (DUBs) plays an important role in orchestrating DNA damage response as well as DNA repair processes. Deregulated ubiquitination and deubiquitination could lead to genome instability that in turn causes tumorigenesis. Recent TCGA study has further revealed the connection between mutations in alteration of DUBs and various types of tumors. In addition, emerging drug design based on DUBs provides a new avenue for anti-cancer therapy. In this review, we will summarize the role of deubiquitination and specificity of DUBs, and highlight the recent discoveries of DUBs in the modulation of ubiquitin-mediated DNA damage response and DNA damage repair. We will furthermore discuss the DUBs involved in the tumorigenesis as well as interception of deubiquitination as a novel strategy for anti-cancer therapy.

## Background

Genomic integrity is constantly challenged by DNA lesions produced as by-products of normal cellular metabolism, DNA replication or induced by radiation and toxic environmental chemicals. DNA damage could lead to detrimental effects on DNA replication and transcription, ultimately generating mutations and chromosomal aberrations that could contribute significantly to tumorigenesis. Upon DNA damage a series of guardian events occur, including the cellular recognition of DNA damage lesion site, initiation and amplification of DNA damage signal to activate DNA damage checkpoint function and activation of various type of DNA damage repair pathways are orchestrated by posttranslational modification, especially protein ubiquitination and deubiquitination, which preserve the genomic integrity.

Ubiquitination, a posttranslational modification covalently attaching ubiquitin to targeted proteins, determines or alters protein’s biological activity, stability or subcellular localization. Unlike the proteolytic regulation, a variety of DNA damage signaling modules are regulated by non-degrading ubiquitin-chain that result in the recruitment of DNA damage proteins to the damage site and activation of protein function. Like the balance of phosphorylation events by the phosphatases, the ubiquitination is counteracted by deubiquitinases.

Deubiquitinating enzymes (DUBs), proteases that reversely modify proteins by removing ubiquitin or ubiquitin-like molecules or remodeling ub-chains on target proteins, have recently be regarded as crucial regulators of both the ubiquitination-mediated degradation and other functions. Therefore, DUBs have a great influence on many biological processes and cellular pathways, including DNA damage response and DNA repair pathways. Thus, exploration of the in-depth mechanism by which DUBs regulate DNA damage response and DNA repair could provide new strategies for anti-cancer therapy.

## General roles of DUBs and DUBs specificity

Ubiquitination, the process in which ubiquitin (Ub) that conjugate ubiquitin to targeted proteins through a cascade composed of E1, E2 and E3 enzymes, plays a vital role in multiple biological processes [1]. Ubiquitin contains seven lysine residues in total 76 amino acids and can form poly-ubiquitin chains of eight different linkages (K6, K11, K27, K29, K33, K48, K63, and Met1), as well as mixed and branched chains [2]. Distinct linkage types result in different chain conformations and display various functions such as protein degradation, localization or protein–protein interactions. For instance, protein degradation through the ubiquitin–proteasome system is mainly mediated by K6, K11, K27, K29, and K48 linked polyubiquitin chains [3]. However, K63 polyubiquitin chains are mainly contributed in the lysosomal pathway and endocytosis, DNA-repair, and signal transduction [4]. Besides, linear chains mediate NF-κB and Wnt signaling, cell death and appear to be required for angiogenic processes [5]. Single ubiquitin molecule could be conjugated to the substrate and is involved in the control of endocytosis, intravesicular transport, transcriptional regulation, DNA replication, and repair [6].

The reversal modification of adding ubiquitin to targeted proteins relies on deubiquitinating enzymes (DUBs), which catalytically cleave single Ub or poly-ubiquitin chains from proteins. The human genome encodes approximately 100 potential DUBs which can be classified into six families: ubiquitin-specific proteases (USPs), ubiquitin COOH-terminal hydrolases (UCHs), ovarian tumor proteases (OTUs), Josephins, the JAB1/MPN/MOV34 family (JAMMs) and motif interacting with Ub-containing novel DUB family (MINDYs) [7]. USPs, UCHs, OTUs, Josephins and the newly identified MINDYs families belong to thiol proteases, while the sixth, JAMMs, are Zn^2+^ metalloproteases [8].

## Main roles of DUBs

**Fig. 1:**
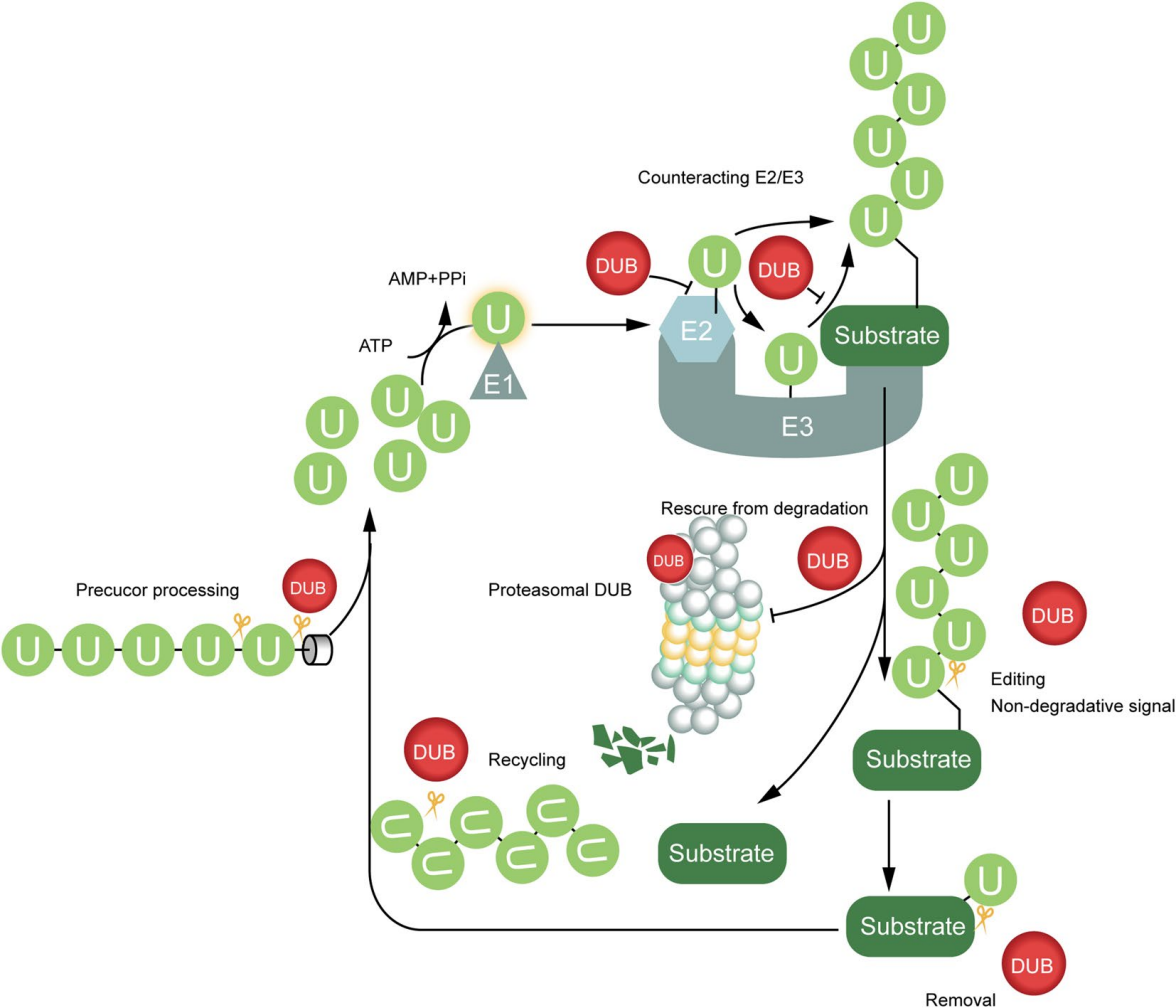
Main roles of DUBs. Deubiquitination is involved in counteracting the ubiquitin cascade, including inhibiting E2 ubiquitin conjugating enzymes and E3 ligases. Proteasome related DUBs help to prevent degradation of ubiquitin chains of proteins treated. Lysosome-associated DUBs play crucial roles in receptor degradation and recycling. Alternatively, DUBs can remove or edit ubiquitin chains to change non-degradation ubiquitin signals. After releasing ubiquitin chains from proteins, DUBs are also responsible for the generation of free ubiquitin from ubiquitin precursors and the release of ubiquitin from unanchored isopeptide-linked ubiquitin chains into ubiquitin pool

The mechanism of protein degradation mediated by ubiquitin has been studied in depth, meanwhile, growing evidence reveals the non-proteolytic roles of ubiquitin modification. Here we will summarize the main roles of DUBs (Fig. 1).

### Counteracting the ubiquitin cascade

#### Modulating E2 activity

Generally, DUBs could inhibit ubiquitination by interfering with the formation and the reactivity of the E2-Ub intermediate. This is a mechanism that couples the opposing activities of the ubiquitination machinery in which DUBs maintain and modulate the dynamic balance of the ubiquitin–proteasome system catalytically or non-catalytically.

Ataxin-3, a DUB associated with Machado–Joseph disease, was reported to reduce the self-ubiquitination of parkin, a familiar form of Parkinson disease-associated E3 ubiquitin-ligase [9]. Intriguingly, Ataxin-3 is unable to remove pre-assembled ub-linkage on Parkin, but can regulate the formation of newly assembled Ub conjugates on Parkin by interacting with Parkin’s E2 conjugating enzyme UbcH7 in a Parkin-depend manner [10]. The temporary formation of E2-parkin-Ataxin-3 complex contributes to the stabilization of E2 and Parkin interaction, impeding the dissociation of the uncharged E2 which can be recharged by E1, meanwhile diverting the Ub from the E2-Ub thioester conjugate onto Ataxin-3 itself, and away from parkin.

OTUB1 has recently emerged as a unique DUB that binds and inhibits several classes of E2s, including Ubc13 and UbcH5s, without reflecting DUB activity per se [11]. OTUB1 was shown to directly bind the Ub thiolester Ubc13 intermediate (Ubc13 ~ Ub). The N-terminal residues of the OTU domain in OTUB1 are required for binding to UBC13 ~ Ub and this interaction is facilitated by the binding of a free Ub to a second site in OTUB1, resulting in allosteric change in the OTU domain and the formation of a ubiquitin-binding helix in the N-terminus which increase its affinity for UBC13-Ub. By binding to OTUB1, UBC13-Ub could neither transfer ubiquitin nor bind to E3 ligase. Similarly, by predominately binding to “charged” UbcH5b, OTUB1 was concluded to function as an E2 inhibitor, reflected in preventing the auto-ubiquitination of the E3 ligase TRAF6.

USP7 is a deubiquitinating enzyme found in all eukaryotes that catalyzes the removal of ubiquitin from specific target proteins such as Mdm2, ICP0, and p53 [12]. USP7 could interact and forms a complex with an E2 ubiquitin conjugation enzyme, UbE2E1, requiring the N-terminal ASTS sequence of UbE2E1. As a result of binding, UbE2E1-mediated ubiquitination was attenuated via the ASTS motif within its N-terminal extension and the catalytic domain of USP7. Inactivation or disruption of the interaction between USP7 and UbE2E1 could lead to UbE2E1 destabilization as well [13].

#### Counteracting E3s

Many DUBs are associated with E3 ligases in pairs or complexes. DUBs co-regulate with E3 ligase partner to fine-tune the ubiquitin loading and removal of target proteins, which even refer to the E3 ligases when they could be self-ubiquitylated. The DUBs could be treated as prey when they are ubiquitinated by its E3 ligase partner or others.

USP10 is one of DUBs which regulate the stability of p53 both under physiological condition and in response to DNA damage with its E3 partner Mdm2. The main role of USP10 is to maintain the stable level of p53 in cytosol [14]. However, following DNA damage, part of USP10 translocate into nucleus to deubiquitylate p53 and thus boost p53 activation. With another E3 ligase partner Huwe1, USP10 appears to modulate the degradation of TATA-binding protein (TBP) during myogenesis [15]. In myoblasts, Huwe1 and USP10 co-operate to keep the homeostasis of TBP. Upon muscle differentiation stimulation, increased Huwe1 and declined USP10 lead to TBP ubiquitination and its proteasomal degradation.

A typical characteristic of E3 ligases is the ability of self-ubiquitination. Many E3 ligases catalyze their own ubiquitination in intermolecular or intramolecular mode, leading to degradation or non-proteolytic outcomes such as activity regulation. DUBs can reverse these ubiquitination events, modulating E3 ligase stability or activity and dynamically controlling the abundance of downstream substrates.

USP15 deubiquitylates autoubiquitinated Mdm2 to regulate p53 function and cancer-cell survival, while the stabilized Mdm2 negatively regulates T cell activation by targeting the transcription factor NFATc2 [16]. USP7 deubiquitinates ubiquitinated (by itself or external ligase such as E6AP) RING1B ligase of the polycomb complex [17]. Ataxin-3 interacts with monoubiquitinated CHIP and limits the length of the poly-ubiquitin chain of the target protein attached by CHIP. After this fine-tuned ubiquitylation is accomplished, Ataxin-3 removes the single ub from CHIP to terminate their interaction [18]. SMURF1, a Nedd4 family of HECT ubiquitin ligases, is self-ubiquitinated through its intrinsic HECT E3 ligase activity and marked a degradation signal, which is antagonized by USP9X via interacting with SMURF1 through the second WW domain of SMURF1 and the carboxyl terminus of USP9X [19].

Mdm2/USP7 and Ro52/USP4 are two E3/DUB pairs which are transregulated by each other. [20, 21]. When the substrate proteins are not required for degradation, the E3 ligases will be auto-ubiquitylated and their DUB partners are responsible for their stabilization. Conversely, USP4 can be ubiquitylated by Ro52 and subsequently degraded.

### Assisting degradation machinery

#### Proteasomal route related DUBs

POH1/PSMD14/Rpn11 is a constitutive stoichiometric component in the 26S proteasome “cap”-19S regulatory particle (RP) and is essential for the RP’s assembly. POH1, belonging to metalloproteases subfamily JAMMs, is responsible for the hydrolysis of ub-chains before proteins are unfolded and degraded [22]. However, before deubiquitination of the substrate by POH1, two other DUBs UCH37 and Ubp6/USP14 antagonize protein degradation by trimming ubiquitin chains from the distal end of the chain leading to a decreased affinity of the protein for the proteasome [23, 24]. Unlike UCH37, USP14 not only removes single ubiquitin from Ub-chain but also bi- or tri-Ub, it can also preferentially remove ubiquitin chains en bloc from substrates with multiple ubiquitinated sites [25]. Besides, Ubp6 was also shown to stabilize the substrate via allosteric interference with the binding of the incoming substrate with the proteasome [24].

#### Endocytic pathway related DUBs

Research in endocytic pathways, especially the largely focused lysosomal degradation of cell-surface receptors, pointed out two DUBs, AMSH and USP8/UBPY [26, 27]. These two DUBs both localize to sorting endosomes through interactions with the endosomal sorting complex required for transport (ESCRT) components of the ESCRT machinery, mainly the ESCRT-0 component signal transducing adaptor molecule (STAM) and the ESCRT-III charged multivesicular body proteins (CHMPs) [28]. While both K63-specific DUB AMSH and non-ub-chain specific USP8 balance the receptor degradation and recycling, exhibiting negative regulation of lysosomal sorting, the roles of AMSH and USP8 are worthy of digging at depth [29]. AMSH and USP8 showed a positive role in the downregulation of protease-activated receptor 2 and additionally, USP8 exhibits pleiotropic effects considering its regulatory role in ESCRT-0 and receptors per se [30, 31].

### Maintaining ubiquitin homeostasis

Maintaining ubiquitin homeostasis includes the generation of Ub precursors from encoded genes, the trim of Ub precursors to free Ubs, the disassembly of polyubiquitin chains from proteins, and the recovery of Ub from chains and other inadvertently trapped Ub derivatives.

In mammals, four Ub precursors encoded by different genes are UBA52, UBA80, L40 and S27A, of which the former two are C-terminal single Ub fused to a ribosomal protein (Ub-RPs), and the rest two are Ub polymers linked in “a head to tail” mode followed by various amino acids in C-terminus (polyUbs). USP5 and Otulin/Gumby/FAM105b preferentially catalyze polyUbs both co- and post-translationally, while UCHL3, USP9X and USP7 are found to be the main enzymes of Ub-RPs in charge in a form of post-translational modification [32]. USP5 is the major DUB which releases ubiquitin from unanchored isopeptide-linked ubiquitin chains, through the ZnF-UBP domain that recognizes the free C-terminus of ubiquitin [33].

## Specificity of DUBs

### Cleavage specificity

#### Ub recognition

The primary Ub binding site that DUB catalytic domains possess has substantial interactions with the distal Ub in a poly-ub chain mainly through Ile44 patch, with different interacting surfaces among DUB subfamilies [34]. The C-terminus of the distal Ub forms a firmly held stretch from the binding site into the DUB catalytic center, allows DUBs to catalyze and distinguish Ub from other ubiquitin-like molecules (ULMs). The C-terminal sequence of Ub (Leu71, Arg72, Leu73, Arg74, Gly75, Gly76), is what makes it different from those of ULMs, and among these six amino acids, Arg74 and Gly75 are crucial for ubiquitin recognition by DUBs [35]. Due to possessing the same C-terminal sequence of Ub, a ULM interferon-stimulated gene 15 (ISG15) could be recognized by some DUBs [36]. However, USP18 can only cleave a linear fusion of ISG15 but not of ubiquitin, suggest the existence of different specify levels of DUBs [37].

#### Linkage preference

**Fig. 2:**
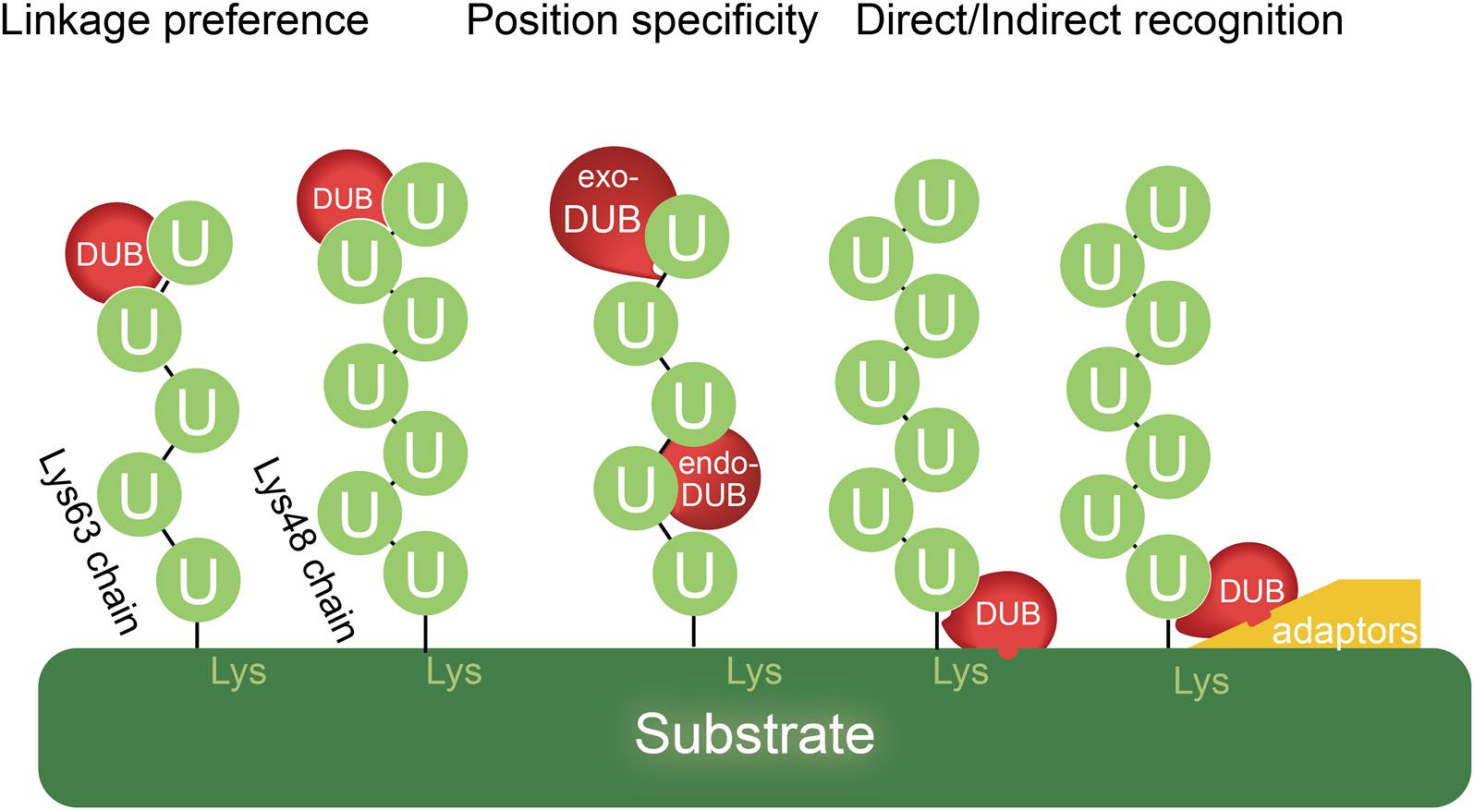
Specificity of DUBs. The recognition and cleavage of ubiquitin chains requires multiple layers of specificity, including the distinguish of ubiquitin from ubiquitin-like molecules, the ubiquitin linkage preference, the position of cleavage site and the recognition of targeted proteins with or without the assistance of adaptors or scaffolds

As the different conformations of diverse linkage types and chain lengths determine the Ub signals and thereby the fate of target proteins, it is not surprising that some DUBs have linkage specificity in the deubiquitylation reaction (Fig. 2). Most OTU or JAMM protease members show inherent specificity. For instance, OTUB1 has a striking specificity for K48-linked chains thus protecting the substrates from degradation and AMSH, AMSH-LP and BRCC3 prefer to cleave non-degradative K63-chains, while the OTULIN preferentially cleaves linear Ub chains [11, 38, 39]. On the other hand, other DUBs like USP family members display little linkage selectivity [40].

#### Positioning specificity (exo-/endo-/mono-DUB)

Ubiquitin chains can be cleaved from the distal part (exo) or internally (endo). USP14, as mentioned above, cleaves K48-linked chains from the distal end only (exo-activity), generating mono-ubiquitin [41]. In comparison, endo-cleavage could be observed in those non-degradative ub-chains by DUBs such as CYLD and AMSH-LP [42, 43]. The positioning specificity could be explained based on DUB’s structure difference. USP14 encompasses a finger subdomain which contacts up to 40% of the distal ubiquitin and blocks access to K48 or K63, allowing USP14 to bind to the distal end of an ubiquitin chain, but not to internal linkages. However, CYLD, due to the lack of the fingers subdomain, allows access to K63 [44, 45]. The cleavage of the first Ub molecule of a poly-ub chain requires DUBs with lower specificity of ub-chain linkage such as UCH subfamily members UCHL3, considering its role in processing precursor Ub [32]. Similarly, processing of monoubiquitin also requires non-specific DUBs which could adjust in their proximal binding site and also recognize the protein substrate [46]. The change of one chain type to another type, which would detour the fate of the substrate, would be easier for the protein with a proximal Ub left on.

### Substrate protein recognition

Apart from linkage and positioning specificity, another feature of DUBs is substrate selectivity. As a consequence, many DUBs are found associated with substrates directly through the binding domains, or indirectly via adaptors and scaffolds.

Some DUBs display affinity for the ubiquitinated protein directly through their protein interaction domains. Crystal structure analysis showed that USP7 binds to its substrate p53 and its inhibitory interactor Epstein–Barr nuclear antigen 1 (EBNA1) protein through the same pocket but the former binding partner p53 exhibit weaker contacts with USP7 [47, 48]. Further functional studies indicated that EBNA1 binding to USP7 inhibits its interaction of p53 and protects cells from apoptotic challenge by lowering p53 levels [12].

Adaptors or scaffolds could facilitate the association between DUBs and substrates. Adaptor protein p62 binds to CYLD and recruits it to TRAF6 [49]. NEMO, another potential adaptor of CYLD, directly binds CYLD and associates with various IKK regulators, such as RIP1 and TRAF2 [50]. OTUD4, rather than being a DUB, acts as a scaffold for USP7 and USP9X, two DUBs that act directly on the DNA demethylases such as ALKBH2 and ALKBH3 [51]. Functionally, the loss of OTUD4, USP7, or USP9X in tumor cells leads to significantly increased sensitivity to alkylating agents. The translation initiation factor 3f (EIF3F) is recruited to activate Notch on endocytic vesicles by the Deltex1 serving as a bridging factor. Notch couldn’t be processed by the gamma-secretase until it’s deubiquitinated by EIF3F [52].

## DUBs and genomic integrity

### DNA damage response main components and signaling

**Fig. 3:**
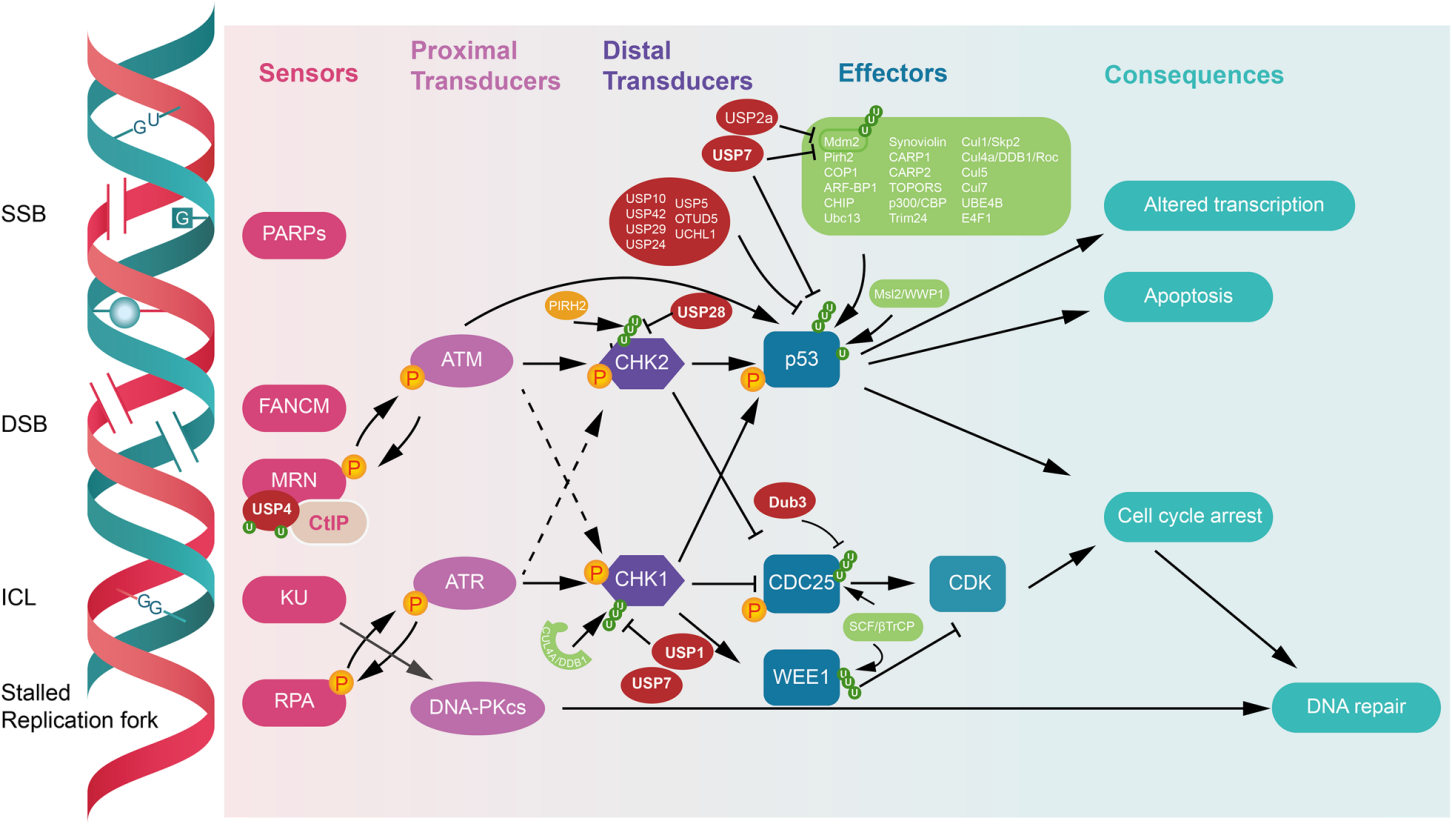
DUBs that modulate the key factors of the DNA damage response leading to different cell fates. USP4 was found to interact with one of the DNA damage sensors MRN complex and the DNA end resection factor CtIP and interfered with CtIP and MRN binding, thus impairing DNA end resection and HR [56]. USP1 and USP7 are reported to be involved in deubiquitination and stabilization of Chk1. USP28 forms a complex with PIRH2 and CHK2 and antagonizes PIRH2-mediated polyubiquitylation and proteasomal degradation of CHK2. Several deubiquitinating enzymes to date have been identified targeting p53 which will be discussed in this review. These DUBs can target p53 directly or indirectly by regulating the E3 ligase Mdm2. DUB3 mediates deubiquitination of CDC25A, preventing CDC25A degradation during the G1/S and G2/M phases, promoting cell-cycle progression [57]

In the face of the continuous threat from both exogenous and endogenous genotoxic insults, cells generate a complex network to maintain the genomic integrity, which is vital for various aspects of organism physiology, ranging from homeostasis to cancer prevention. DNA damage response (DDR), which includes surveillance proteins monitoring and detecting DNA damage, activating cell cycle checkpoints and ensure the effective DNA damage repair [53]. The checkpoint response can repair the damaged DNA before it passes on through mitosis, or make the decision of apoptosis if the damage is too difficult to repair [54]. DDR coordinates DNA repair with vital cellular functions to determine the fate of the cell after DNA damage [55]. As the fact that ubiquitination plays a prominent role in DDR, it could be expected that DUBs also serve as crucial regulators in DDR and DNA repair pathways (Fig. 3).

Various types of DNA lesions including DNA single- and double-strand breaks (SSBs and DSBs) are generated all the time in cells. Sensors such as MRN complex, Ku70/Ku80 heterodimer (KU) and PARPs are activated in response to DSBs (the former two) and SSBs [58]. FANCM, act as the sensor of interstrand crosslink (ICL)-induced checkpoint response [59]. RPA binds to regions of exposed single-stranded DNA (ssDNA) in lesion area and the following events are the recruitment of ATM and ATR-ATRIP mediated by MRN and RPA respectively, the subsequent activation of the downstream pathways [60]. KU recruits DNA-PKcs to form the catalytically active DNA-PK holoenzyme in the canonical non-homologous end joining (NHEJ) repair pathway [61]. On the other hand, MRN initiates homologous recombination (HR) [62].

Once activated, the cell-cycle checkpoint kinases CHK1 and CHK2 trigger the DNA damage signaling cascade to extend, gathering downstream effectors such as the p53 or the CDC25 and WEE1 [63]. Consequently, cyclin-dependent kinase (CDK) activity is inhibited, stalling cell cycle progression from G1 to S (the G1/S checkpoint) or from G2 to M phase (the G2/M checkpoint) [64]. The DDR thus masterminds a variety of events including the altered transcriptional program and the contemporarily arrested cell cycle, thereby facilitating repair of the DNA lesions. When DNA damage is too severe to be repaired, the fate of the damaged cell is apoptosis or senescence [65].

USP4 was found to interact with the DNA end resection factor CtIP and MRN complex via its C-terminal insert region and promoting the binding of CtIP/MRN by contracting its own ubiquitylation, which interfered with CtIP and MRN binding, thus impairing DNA end resection and HR [56]. UCH37, as previously mentioned, is a 19S regulatory particle related DUB as well as a component of INO80 chromatin-remodeling complex which is known to directly associate with DSB ends and is required for DSB end resection and overall DSB repair [66, 67]. Interestingly, Ku70 was found to function as a DUB to stabilize Mcl-1 by directly interacting with Mcl-1 via its C-terminus, which is required and sufficient for deubiquitination and stabilization of Mcl-1, leading to suppression of apoptosis [68].

USP1 and USP7 are reported to be involved in deubiquitination and stabilization of Chk1 [69, 70]. USP7 was also shown to regulate other DDR proteins such as Claspin, an adaptor protein activated by Chk1 in the ATR–Chk1 pathway [71]. Importantly, the USP7 catalytic mutant is in a mono-ubiquitinated form, suggesting it is self-regulated by its hydrolase feature. Additionally, USP29 and USP20 were found to be other DUBs for Claspin, [72, 73].

The E3 ligase PIRH2 interacts with and ubiquitinates CHK2 dependent on its phosphorylation status. USP28 forms a complex with PIRH2 and CHK2 and antagonizes PIRH2-mediated polyubiquitylation and proteasomal degradation of CHK2 [74].

The ubiquitin modification of p53 is much complicated than that of other DDR components. Several E3 ligases target p53, of which Mdm2 plays a major role both in controlling basal levels of p53 in normal unstressed cells and in response to stress conditions [75]. Other E3 ubiquitin ligases identified include COP1, Pirh2, ARF-BP1, MSL2, and Parc [76–78]. On the other hands, several deubiquitinating enzymes to date have been identified targeting p53. These DUBs can target p53 directly or indirectly by regulating the E3 ligase Mdm2. USP7 was the first DUB identified to target p53 and Mdm2 for deubiquitination [79]. USP2a specifically deubiquitinates Mdm2 and MdmX [80]. In contrast to USP7 and USP2a, USP10 specifically deubiquitinate p53 because knockdown of USP10 in HCT116 p53−/− cells does not cause Mdm2 reduction [14]. Importantly, USP10 can be phosphorylated by the ATM kinase, leading to its stabilization and nuclear translocation. Similarly, USP42 is a p53-specific deubiquitinase and plays a role in DNA damage-induced p53 stabilization [81]. USP24 is required for p53 stabilization in unstressed cells, as well as for p53 stabilization and PUMA activation after DNA damage [82]. Both OTUD5 and USP29 are required to be p53-dependent transcriptionally induced to stabilize p53 in response to DNA damage stress [83, 84]. Additionally, USP5 indirectly regulates levels of p53, while UCHL1 forms a complex with p53/p14 (ARF)/Mdm2 p53 binding protein homolog in the mouse [85, 86]. Recently, CYLD was shown to promote DNA damage-induced p53 stabilization and activation in epithelial cells and inhibit chemical carcinogen-induced intestinal and skin tumorigenesis [87]. Taken together, the varying actions of these deubiquitinases allow for dynamic p53 regulation in a context-dependent manner.

DUB3/USP17 mediates deubiquitination of CDC25A, preventing CDC25A degradation by the proteasome during the G1/S and G2/M phases promoting cell-cycle progression [57]. USP50 was identified as an interacting partner of HSP90. In response to DNA damage, USP50 accumulates in the nucleus and may act through an HSP90-dependent mechanism to counteract CDC25B mitotic inducing activity and prevent Wee1 degradation, thereby repressing entry into mitosis following activation of the DNA damage checkpoint [88].

### DNA damage repair

**Fig. 4:**
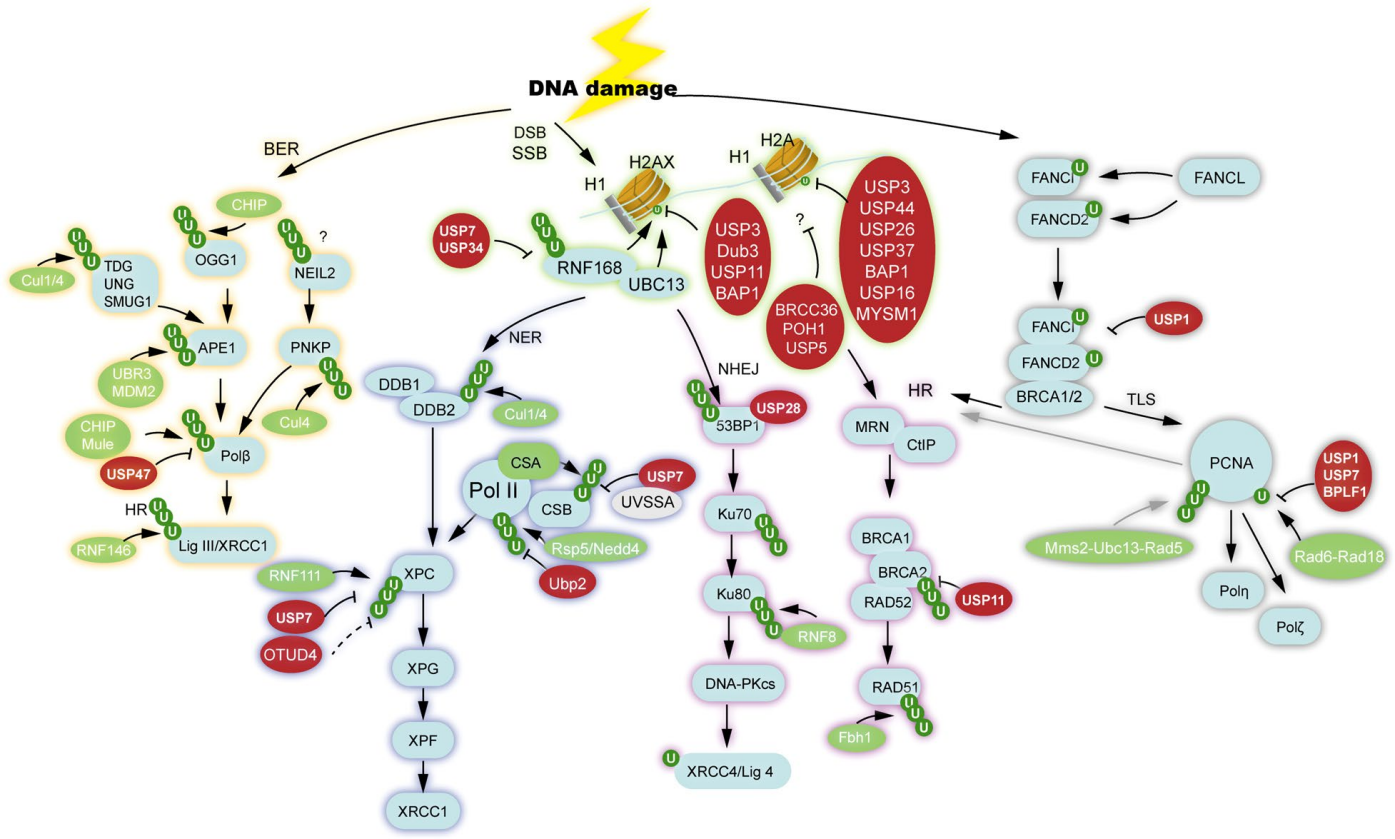
DUBs that regulate major DNA damage repair pathways, including the modification of histones (with *green outer* glow), base excision repair (with *yellow outer* glow), nucleotide excision repair (with *violet outer* glow), homologous recombination and non-homologous end joining (with *pink outer* glow), and inter-strand crosslink damage repair including Fanconi anemia pathways and translesion synthesis (with *grey outer* glow)

DNA may be modified resulting from numerous genotoxic agents such as ultraviolet in the form of single-strand breaks (SSBs) and/or double-strand breaks (DSBs) [89]. UV-induced damage also can result in the production of pyrimidine dimers and the formation of covalent cross-links [90]. Rapid and well-organized repair machinery composed of sensors and repair proteins are responsible for removing these lesions thus maintaining genomic integrity. Major repair pathways include base excision repair (BER), mismatch repair (MMR), nucleotide excision repair (NER), homologous recombination (HR), non-homologous end joining (NHEJ), and translesion synthesis (TLS) (Fig. 4) [91].

#### Single strand break

Since only one of the double strands of DNA is defective, the other strand could be used as a template. Taking advantage of this situation, several excision repair mechanisms exist, among which the BER repairs small base lesions while NER deals with bulky helix-distorting lesions.

The initial step of BER is performed by DNA glycosylases, which scan along the DNA backbone to recognize and remove defective bases and form apurinic/apyrimidinic (AP) sites. These AP sites are then processed by AP endonuclease 1 (APE1) and DNA polymerase β (Pol β) to leave a single strand break and synthesize a new, no-error nucleotide. The final nick-sealing work is accomplished by DNA ligase IIIα (Lig III) along with its cofactor X-ray cross-complementing protein 1 (XRCC1) in short-patch BER. DNA ligase I ligates the break in long-patch BER [92]. Besides, endonuclease VIII-like proteins (NEIL1-3) have been identified as new human DNA glycosylases, with similar mistake elimination function but different lesion preferences [93].

USP47 is the major enzyme involved in deubiquitylation of Pol β. USP47 stabilizes the cytoplasmic Pol β which will relocate to the nucleus in DNA damage pathway. Knockdown of USP47 decreased the level of Pol β which defect the BER pathway, leading to accumulation of DNA strand breaks induced by DNA damaging agents [94].

NER repairs bulky DNA base adducts and ultraviolet light-induced lesions. NER can be divided into two main pathways based on the damage recognition mechanism: global genome repair (GG-NER) and transcription-coupled repair (TC-NER). The two pathways share the same processes in incision, repair, and ligation. DDB1-DDB2/XPE and XPC/RAD23 complexes are responsible for damage detection in GG-NER [95].

DDB2, associates with DDB1, to recruit XPC to chromatin, and also facilitates the recruitment of cullin 4A/B-RING ubiquitin ligases which ubiquitinate various acceptor proteins including DDB2 and XPC [96]. When RNA polymerase II (RNAP II) stalls upon encountering a DNA lesion during transcription, TC-NER is activated and RNAP II is ubiquitinated and dislocated from chromatin. Recognition of damage is dependent on CSB (ERCC6), which associates with RNAP II and recruits CSA (ERCC8) to the lesions, the latter serves as E3 ligase of CSB in the CSA-CUL4A complex [97]. Reversibly, USP7 regulates NER targeting XPC protein and preventing XPC protein from undergoing UV-induced and VCP/p97 regulated proteolysis [98]. Furthermore, USP7 and UVSSA protein couple and counteract CSA-dependent degradation of CSB to allow sufficient time for CSB to perform its function in TC-NER when RNAP II is remodeling [99]. Proteolysis of damage-induced RNAP II is tightly regulated by both E3 ligases and DUB. In yeast, it has been shown that the degradation associated K48-linked ub chain is generated in 3 steps. Rsp5 E3 (NEDD4 in mammals) catalyzes K63-linked ub chain which is trimmed by a DUB Ubp2 resulting in the mono-ubiquitination of RNAP II, prompting a second E3 ligase Elongin/Cullin 3 complex to generate K48-linked ub chains [100].

#### Double-strand break

HR and NHEJ are two major DSB repair pathways. HR repair generates error-free strands by acquiring genetic information from sister chromatids, whereas NHEJ may lead to mutagenesis by ligating two broken ends directly, in which process the loss of the nucleotide in DSB may cause deletion and joining of non matching ends may cause insertions or translocations [101]. Increasing evidence has demonstrated the important role of DUBs in mediating the DSB repair pathways.

Post-translational modifications of histone, especially phosphorylation of H2AX by ATM and sequential recruitment of MDC1, is the key initial event in DSB repair [102]. Phosphorylated MDC1 by ATM recruits E3 ligase RNF8 to add K63-ub chains to H1, forming a binding site for RNF168 to H1 [103]. Then RNF168 is ready to induce K63-/K27-ubiquitination on H2A, which in turn enhance the recruitment of RNF168 [104]. Besides, ubiquitination of H2B by RNF20–RNF40 is demonstrated to be crucial in response to DSB, as this ubiquitination event is important for the formation of open and biochemically accessible chromatin fiber that is conducive to DNA repair [105]. H1 and H2A may not be the respective substrates for RNF8 and RNF168 at DSB lesions. Recent studies have revealed that the polycomb molecule L3MBTL1 and the lysine demethylase JMJD2A are also substrates of RNF8 [106, 107]. RAP80 is a key factor at ubiquitinated structures on chromatin surrounding DSB sites. RAP80 facilitates the recruitment of BRCA1 to DSB sites as a scaffold molecule but the BRCA1-RAP80 complex limits nuclease accessibility to DSBs, thus preventing excessive end resection and potentially deleterious HR [108]. RAP80 also helps to recruit BRCC36, which regulates the NHEJ repair [109]. 53BP1, a key factor in NHEJ pathway, interacts tightly with nucleosomes containing both H4K20me2 and RNF168-dependent ubiquitinated histone H2A [110]. 53BP1 promotes the NHEJ pathway via the inhibition of BRCA1 recruitment, the recruitment of RIF1 and REV7 (anti-DNA end resection factors) and the recruitment of Artemis nuclease through PTIP [111].

DUBs of H2A and H2AX are partially shared. USP3, Dub3, USP11 and BAP1 show their DUB ability in H2AX-ub, while USP3, USP44, USP26, USP37, BAP1, USP16, and MYSM1 are DUBs which remove ubiquitin or ubiquitin chains from H2A. USP44 also can deubiquitinate H2B-Ub [112–115]. On the other hand, the stability of RNF168 is sustained by DUB USP34 and USP7. Recently, OTUB2 was suggested to target L3MBTL1 and K 63-linked ubiquitin chains to counteract the function of RNF8 and thus enhanced recruitment of 53BP1 and RAP80 [116]. USP11 was shown to interact with and deubiquitinates BRCA2 and as well counteracts RNF4-induced SUMO-ubiquitin hybrid chains, suggesting the pleiotropic roles at DSBs sites [117]. USP28 was shown to bind 53BP1, but only minor DDR defects were observed in USP28-depleted cells, suggesting its minor role in DSB repair. [118]. UCH37 was reported to regulate DSB resection and repair by HR pathway through stabilizing nuclear factor related to Kappa-B-binding protein (NFRKB) [66].

There are some DUBs found to be crucial in removing ub/ub-chains at DSB sites without clear substrates such as BRCC36, POH1, and USP5, which antagonize the K63-linked polyubiquitin conjugates at damage sites [109, 119].

#### Interstrand crosslink

ICLs are thought to be a highly toxic type of DNA damage which prevent transcription and replication. Defective repair of DNA of ICLs is a key feature of Fanconi anemia (FA). FA pathway is now thought to involve the coordination of HR, NER and TLS. There are currently 15 known genes (FANCA to FANCP) whose bi-allelic mutations yield FA [120].

Central to FA pathway is the monoubiquitination of FANCD2 (K-561) and FANCI (K-523) by the FA core subunit FANCL [121, 122]. This monoubiquitination is stimulated by DNA damage and it sends the signal to other FA proteins such as nucleases FANCP (SLX4) and FANCQ (XPF), and downstream repair factors like FANCJ (BRIP), FANCN (PALB2), FANCD1 (BRCA2), and FANCO (RAD51C) [120].

USP1 was one of the first ubiquitin hydrolases characterized as a key player in ICL repair pathways. USP1, the major DUB of FANCD2 and FANCI, inactivates these two proteins mediated by the USP1-activating factor UAF1 once DNA damage repair is finished [123].

DUBs indeed affect many other DNA damage repair processes, taking PCNA as an example. Under replication stress, PCNA is monoubiquitinated by the UBE2B-RAD18 and then recruits and activates potential error-prone DNA polymerases. Poly-ubiquitination of PCNA induced by E2 complex UBE2N–UBE2V2 and the E3 ligases HLTF, RNF8 and SHPRH makes it involved in an error-free template switching pathway [124]. USP1 and USP7 are identified as a DUB of mono-ubiquitinated PCNA acting in different cell cycle phases (S-phase and interphase respectively) [46, 125]. Since PCNA is reported to associate with Epstein–Barr virus (EBV) DNA during its replication, an EBV DUB encoded by BPLF1 was found to target ubiquitinated PCNA and disrupts TLS [126].

## DUBs involved in diseases and DUBs targeting therapeutics

Growing evidence indicates germline and somatic mutations, as well as expression frequency alterations of DUBs, are correlated with human disease, ranging from immune diseases to many human cancers.

### DUBs and diseases

Mutations and deletions in CYLD have been reported in Brooke-Spiegler syndrome (BSS), familial trichoepithelioma and malignant transformation [127]. Mutated CYLD disrupted its inhibitory function on NF-kB and HDAC pathways, resulting in the activation of MYB, which plays a vital role in the biology of cylindroma either sporadic or emerged with BSS [128]. Additionally, CYLD has also be linked to immune response through its regulation on Tak1 with E3 ligase Itch, leading to the degradation of Tak1 resulting in the termination of inflammatory necrosis factor signaling [129]. A20 is another negative regulator of NF-kB pathway. A number of studies have reported the deletions or mutations of TNFAIP3 (encoding gene of A20) in lymphomas such as marginal zone lymphoma and Non-Hodgkin’s lymphoma, indicating A20 as a tumor suppressor and immune regulator [130]. Recently, high penetrance heterozygous germline mutations in TNFAIP3 were considered as the cause of an auto-immune related syndrome Haplo insufficiency of A20 (HA20), displaying early-onset systemic inflammation, arthralgia/arthritis, oral/genital ulcers and ocular inflammation. Mutated A20 results in truncated proteins which is defective in inhibit NF-kB pathway, leading to an increased expression of NF-κB-mediated proinflammatory cytokines [131]. BAP1, as mentioned above, could remove ubiquitin from H2A in the complex with ASXL1 [132]. However, recent research revealed a new mechanism of loss of BAP1 contributing to tumorigenesis. By targeting atypical polycomb protein L3MBTL2, BAP1 interacts with and stabilizes L3MBTL2, co-occupying and maintaining H4K20me1 at target gene loci, such as EZH2 locus. Loss of BAP1 leads to reduced L3MBTL2 stability and increased EZH2 transcriptional output in mesothelioma [133]. USP8 gene somatic mutations are found in corticotroph adenomas, which results in pituitary corticotroph adenomas hypersecreting adrenocorticotropin (ACTH) and is the major cause of Cushing’s disease. Mutated USP8 protein is truncated due to the loss of binding site for 14-3-3 protein and gains a higher DUB activity. This leads to increased recycling of its substrate EGFR, which accumulates on plasma membrane and stimulates Pomc gene transcription and increase plasma ACTH levels [134].

Numbers of DUBs are associated with tumors by their alteration in protein expression. For instance, increased expression level of OTUD6B, UCH37, VCPIP1, USP7 and COPS5 are detected in breast cancer [135]. USP6 is considered as an oncogenic protein and overexpressed in primary aneurysmal bone cyst (ABC) and nodular fasciitis by chromosome translocation, and forms fusion proteins with CDH11, TRAP150, ZNF9, OMD, and COL1A1, which result in promoter swapping and transcriptional up-regulation [136]. However, roles of some DUBs are poles apart in different tumor types. In ovarian and prostate carcinoma, USP2 protein is upregulated, whereas in colon cancer, USP2 expression is downregulated [137].

### Therapeutics targeting DUBs

Specific mechanisms of deubiquitinating enzymes in various diseases have been described. Research should be concentrated on discovering an inhibitor on DUB’s enzyme activity or antagonist which binds the substrates for therapy of cancer and other diseases (Table 1).

**Table 1:** DUB inhibitors

Compound	Reported activity/target	Ref.
*Inhibitors containing Michael acceptors*		
12Δ-PGJ2	UCHL3	[139]
15Δ-PGJ2	UCHL1	[139]
G5	Broad spectrum DUB inhibition	[140]
b-AP15	USP14/UCH37	[141]
VLX1570	USP14/UCH37	[141]
AM146, RA-9 and RA-14	USP2a/USP2b/USP5/USP8	[143]
WP1130	USP5/USP9x/USP14/UCHL1/UCHL5	[144]
Eeyarestatin 1	Ataxin-3	[145]
*Small inhibitors*		
AC17	USP14/UCH37	[142]
P022077	USP7	[146]
HBX 41, 108	USP7	[148]
HBX-19, 818	USP7	[149]
HBX-28, 258	USP7	[149]
P5091	USP7	[147]
Cpd 14	USP7/USP47	[150]
P22077	USP7/USP47	[151]
IU1	USP14	[139]
LDN-57444	UCHL1	[152]
LDN91946	UCHL1	[153]
LS1	UCHL3	[156]
PR-619	Broad spectrum DUB inhibition	[157]
15-oxospiramilactone (S3)	USP30	[158]
*Clinical drugs*		
Pimozide	USP1	[154]
Auranofin	proteasome-associated DUBs	[155]

### DUB inhibition by compounds containing Michael acceptors

Compounds containing Michael acceptors such as α, β-unsaturated ketones have the inhibitory effect on some of cysteine DUBs due to the fact that they can potentially form covalent adducts with free thiols in the active site [138]. Cyclopentenone prostaglandins (PGs) of the PGJ2 class, chalcone compounds and other compounds containing Michael acceptors will be discussed here.

UCHL3 was found to be inhibited by Δ12-PGJ2 and UCHL1 by 15Δ-PGJ2 [139]. Chalcone compounds G5 has a broad inhibitory spectrum, whereas another chalcone compounds b-AP15 and its analogue VLX1570 are relatively specific to USP14 and UCH37 [140, 141]. USP14 and UCH37, are also inhibited by curcumin analogue AC17 [142]. UCHL1, UCHL3, USP2 and USP8 were found to be inhibited by AM146, RA-9, and RA-14 which did not inhibit Ataxin-3, A20, BAP1, Otubain 1 or USP7 [143]. WP1130 acts as a partially selective DUB inhibitor for USP9x, USP5, USP14, and UCH37, resulting in downregulation of antiapoptotic and upregulation of proapoptotic proteins, such as MCL-1 and p53 [144]. Eeyarestatin-1 (Eer1) was identified to inhibit p97/VCP-associated DUB activity such as that of Ataxin-3 [145].

### Other small molecule DUB inhibitors

Due to the multifaceted roles of USP7, many inhibitors have been developed targeting USP7, such as P022077, HBX 41,108, HBX-19,818, HBX-28,258, P5091, Cpd 14 and P22077, in which the latter two molecules also inhibit USP47 [146–151]. A small molecule IU1 has been described as specific inhibitor of USP14, only binding the activated USP14 [139]. LDN-57444 is an isatin O-acyl oxime reported to selectively inhibit UCHL1 in a reversible, competitive, and active site-directed manner [152]. Compared to LDN-57444, LDN91946, 3-Amino-2-keto-7H-thieno [2, 3-b] pyridin-6-one derivative, was discovered as moderately potent, non-competitive inhibitors of UCHL1 [153]. Clinical drugs for treating other diseases previously, were found as DUB inhibitors. Pimozide (an anti-psychotic drug) was identified as inhibitors of USP1, and auranofin (a rheumatoid arthritis drug) is a proteasome-associated DUB inhibitor [154, 155]. Benefiting from high-throughput screening studies, LS1 as an UCHL3 inhibitor and PR-619 as a general DUB enzyme inhibitor [156, 157]. Interestingly, the mitochondria-localized DUB USP30 was found to be inhibited by a diterpenoid derivative 15-oxospiramilactone (S3), leading to the increased Mfn1/2 proteins which promote mitochondrial fusion [158].

Of ~100 DUBs, only several DUBs have been investigated for their structures despite identification of a variety of substrates for various DUBs, providing a rationale to open the way for designing small inhibitor molecules. ‘To date only a few of DUB inhibitors such as VLX1570 are in clinical trials for tumor therapy. And no DUB inhibitor is approved for clinical use. Therefore, much work is still required to be accomplished to validate and develop them to the clinic.

## Conclusion

While the impact of DUBs in the regulation of biological function and human diseases have attracted attention in the field for a decade, there are still quite a few aspects that have not been elucidated. Recent systematic screening of DUBs in regulating various cellular processes leads to diverse landscape of DUBs in regulating different pathways. An interesting puzzle needs to be explained is the observation of DUB substrates. At the biochemical level, how the substrate specificity is established for the limited 100 DUBs to face over thousands targeting proteins needs to be understood. Recently, some new findings enhance our knowledge regarding how DUBs interacts with the ubiquitin cascade. Despite the simple view of removal of ubiquitin chain from the substrate, it has been demonstrated that DUBs could modulate the activity of ubiquitin conjugating enzyme and directly counteract E3 ligase activity as well as to assist degradation machinery. Nevertheless, a better classification of 100 DUBs and their mechanism of counteracting ubiquitin cascade needs to be done. Other than conventional biochemical and cell biological dissection of the role of DUBs, more sophisticated protein structural studies could enhance our understanding of the in-depth mechanism of catalysis of deubiquitination and substrate specificity. As more missense mutations are described on DUBs in relation to tumorigenesis and various diseases, the physiological relevance of individual DUB and important mutation sites need to be validated by disease animal model. While a few DUB small molecule inhibitors shed light on anti-cancer therapy, more efforts are needed in drug development. Given our explored impact of DUBs in regulating DNA damage response and repair, it is important to determine the synergistic role of DUBs with current DNA damaging drugs in radiosensitization or chemosensitization of anti-cancer therapy.

## Abbreviations

ABC: aneurysmal bone cyst
ACTH: hypersecreting adrenocorticotropin
AP: apurinic/apyrimidinic site
APE1: AP endonuclease 1
BER: base excision repair
BSS: Brooke-Spiegler syndrome
CDK: Cyclin-dependent kinase
CHMP: ESCRT-III charged multivesicular body protein
DDR: DNA damage response
DSB: double strand break
DUB: deubiquitinating enzyme
EBNA1: Epstein–Barr nuclear antigen 1
EBV: Epstein–Barr virus
Eer1: Eeyarestatin-1
EIF3F: translation initiation factor 3f
ESCRT: endosomal sorting complex required for transport
FA: Fanconi anemia
GG-NER: global genome repair
HR: homologous recombination
ICL: interstrand crosslink
ISG15: interferon-stimulated gene 15
JAMM: the JAB1/MPN/MOV34 family
KU: Ku70/Ku80 heterodimer
Lig III: DNA ligase IIIα
MINDY: motif interacting with Ub-containing novel DUB family
MMR: mismatch repair
NEIL: endonuclease VIII-like protein
NER: nucleotide excision repair
NFRKB: kappa-B-binding protein
NHEJ: non-homologous end joining
OUT: ovarian tumor proteases
PG: prostaglandin
Pol β: DNA polymerase β
RNAP II: RNA polymerase II
RP: 19S regulatory particle
SSB: single strand break
ssDNA: single-stranded DNA
STAM: signal transducing adaptor molecule
TBP: TATA-binding protein
TC-NER: transcription-coupled repair
TLS: translesion synthesis
Ub: ubiquitin
Ub-RP: ribosomal protein
UCH: ubiquitin COOH-terminal hydrolases
ULM: ubiquitin-like molecules
USP: ubiquitin-specific protease
XRCC1: X-ray cross-complementing protein 1
